# Human observer performance on in-plane digital breast tomosynthesis images: Effects of reconstruction filters and data acquisition angles on signal detection

**DOI:** 10.1371/journal.pone.0229915

**Published:** 2020-03-12

**Authors:** Changwoo Lee, Minah Han, Jongduk Baek

**Affiliations:** 1 Center for Medical Convergence Metrology, Korea Research Institute of Standards and Science (KRISS), Daejeon, South Korea; 2 School of Integrated Technology and Yonsei Institute of Convergence Technology, Yonsei University, Incheon, South Korea; US Department of Agriculture, UNITED STATES

## Abstract

For digital breast tomosynthesis (DBT) systems, we investigate the effects of the reconstruction filters for different data acquisition angles on signal detection. We simulated a breast phantom with a 30% volume glandular fraction (VGF) of breast anatomy using the power law spectrum and modeled the breast mass as a spherical object with a 1 mm diameter. Projection data were acquired using two different data acquisition angles and numbers of projection view pairs, and in-plane breast images were reconstructed using the Feldkamp-Davis-Kress (FDK) algorithm with three different reconstruction filter schemes. To measure the ability to detect a signal, we conducted the human observer study with a binary detection task and compared the signal detectability of human to that of channelized Hotelling observer (CHO) with Laguerre-Gauss (LG) channels and dense difference-of-Gaussian (D-DOG) channels. We also measured the contrast-to-noise ratio (CNR), signal power spectrum (SPS), and *β* values of the anatomical noise power spectrum (NPS) to show the association between human observer performance and these traditional metrics. Our results show that using a slice thickness (ST) filter degraded the signal detection performance of human observers at the same data acquisition angle. This could be predicted by D-DOG CHO with internal noise, but the correlation between the traditional metrics and signal detectability was not observed in this work.

## Introduction

Breast cancer is the most commonly diagnosed cancer among women and the second-highest cause of cancer related mortality [1], but its high mortality rate has steadily decreased through advances in diagnostic imaging systems [2]. Mammography systems are widely used for the early detection of breast cancer, but superimposed breast tissue is an impediment to degrade the lesion detection performance. Unlike mammography systems, digital breast tomosynthesis (DBT) systems acquire projection data from a limited data acquisition angle, and thus, the amount of anatomical noise caused by tissue superposition can be reduced significantly, which has led to improved detection performance of in-plane DBT images [3, 4]. However, several imaging parameters (e.g., data acquisition geometries, reconstruction algorithms, kVp, etc. [5, 6]) affect the image quality of in-plane images from DBT systems, and finding an optimal imaging protocol is very important to maximize the signal detection performance.

To optimize the performance of DBT system, image quality assessment plays an important role, and traditional metrics such as the contrast-to-noise ratio (CNR), signal power spectrum (SPS), and exponent *β* value of the anatomical noise power spectrum (NPS) have been used. In previous studies [7–11], CNR and SPS have been used to find the optimal data acquisition angle for the DBT system for various imaging tasks; they considered high CNR and SPS as indicators of better image quality. The *β* value was also used to compare signal detectability between breast imaging modalities [12–14], and they considered a small *β* value to be an indicator of high detection performance.

In filtered back-projection (FBP)-based DBT reconstruction, there are several reconstruction filters that affect spatial resolution and image noise characteristics (e.g., a ramp or the Hanning weighted ramp filter). Previously, many researchers focused on a particular reconstruction filter to assess the image quality of DBT systems by assuming that the various window do not have significant impact on signal detectability [11, 14–19]. However, the effect of reconstruction filters on signal detectability needs to be explored as the anatomical noise generated by different reconstruction filters shows very different background structures in DBT images, which may have different impact on signal detectability. Another filter type called slice thickness (ST) filter was also used in DBT reconstruction to avoid aliasing artifacts [20, 21], and Zhao et al. [22, 23] compared the effects of the ST filter in combination with a ramp and the Hanning weighted ramp filter on signal detection performance using the signal difference noise ratio (SDNR). They concluded that the ST filter provided a high SDNR for a 1 mm diameter signal, although the SDNR did not reflect the effect of background correlation on signal detection performance.

The most desirable way to evaluate the detection performance for the given task is to conduct a human observer study; this is because human is the end user of the medical images who makes the final diagnostic decision. However, conducting a human observer study is time-consuming and expensive. Moreover, a medical imaging system produces several hundreds of images for each patient, which would introduce more inter-observer variability for the same task [24]. To overcome this, mathematical model observers which mimic human observer performance have been proposed [25–27], and several studies have explored how data acquisition angle, reconstruction algorithm, and background variability affect the signal detection performance in DBT images using mathematical observer models [16–18, 28–30]. However, the effect of reconstruction filters and data acquisition geometries on human detection performance has not been studied thoroughly.

The main contribution of this work is to investigate the effects of reconstruction filters and data acquisition angles in DBT systems on the detection performance of human observer. Furthermore, this performance is compared with both model observer performance and the traditional metrics (i.e., CNR, SPS, and *β* values). To accomplish this, we simulated a breast phantom with 30% volume glandular fraction (VGF) and a breast mass with a 1 mm diameter, and in-plane DBT images were acquired using the FBP algorithm. In FBP-based DBT reconstruction, reconstruction filters and data acquisition geometries have more impact on the signal and background statistics than other parameters (e.g., focal spot blur and detector pixel correlation). Thus, we examined signal detectability using three different reconstruction filter schemes and two different data acquisition angles. The human observer study was conducted for the given tasks, and then, human observer performance was compared with that of channelized Hotelling observers (CHO) with Laguerre-Gauss (LG) channels [16–18, 28, 31] and dense difference-of-Gaussian (D-DOG) channels [26, 31, 32]. We measured CNR, SPS, and *β* values to find the relationship between human observer performance and the traditional metrics.

## Materials and methods

### Image generation

#### Simulated breast phantoms

**Fig 1:**
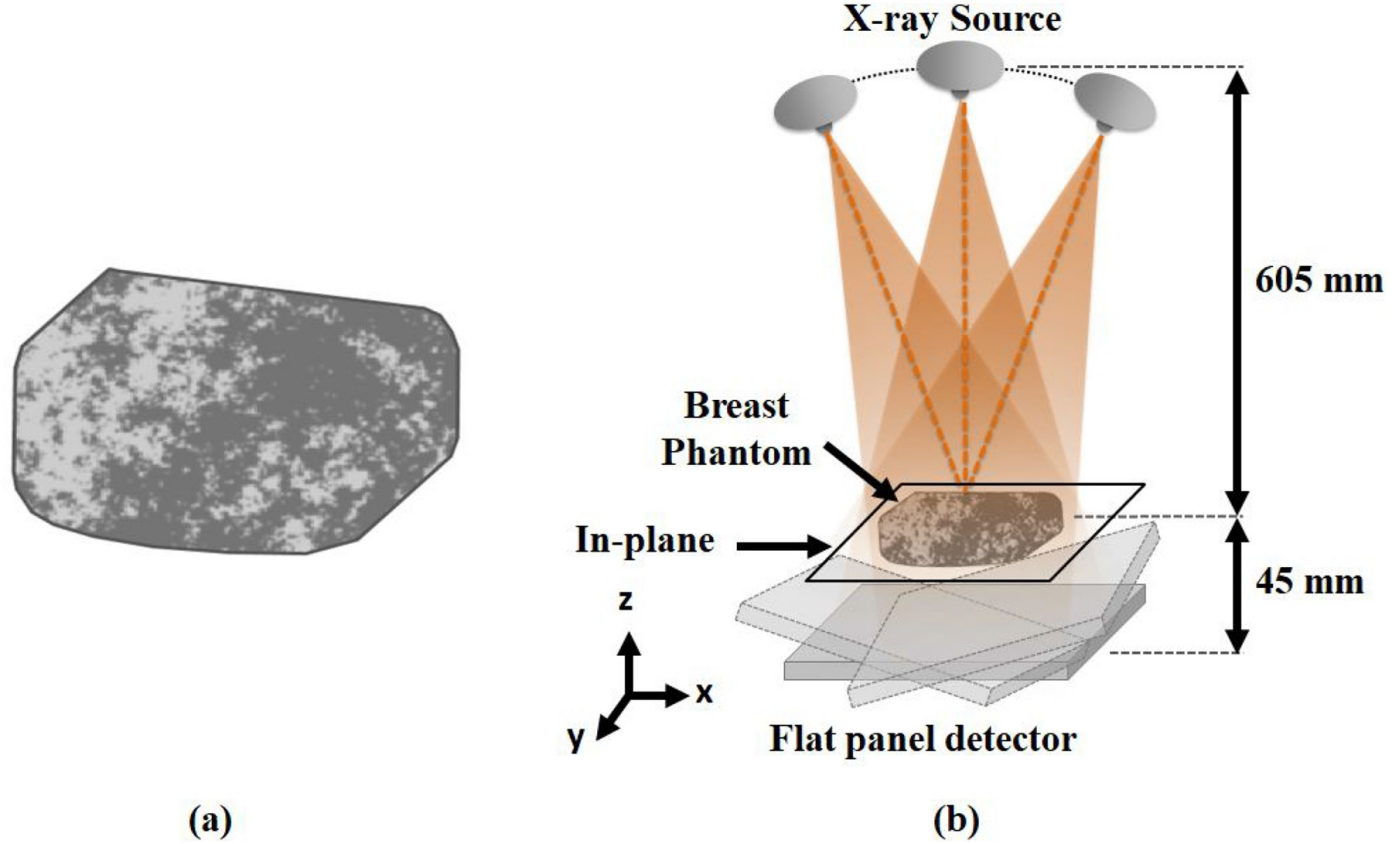
Breast phantom and breast tomosynthesis system geometry. (a) A simulated breast phantom with 30% VGF. The gray and black regions indicate fibro glandular and adipose tissues, respectively. The simulated breast phantoms are displayed in [0 0.1] *mm*^−1^. (b) The system geometry of a digital breast tomosynthesis system with a step-and-shoot mode. The X-ray source and flat-panel detector rotate simultaneously along the arc path. The projection data of simulated breast phantom are acquired within a limited data acquisition angle.

To mimic the morphology of the breast, simulated breast phantoms can be modeled by following the power law spectrum [12, 13, 33]:

$$\begin{array}{c} P\left(f\right)=\alpha /f^{\beta }, \end{array}$$ (1)

where *f* is the three dimensional (3D) radial frequency, *α* is a constant, and *β* is the power law exponent (known to be 3 for mammography) [33]. To generate the simulated breast volumes, we generated a volume with 1024 × 1024 × 1024 voxels of white Gaussian noise and filtered it using the square root of 1/*f*^3^ [15, 34–36]. Note that the zero frequency value of the filter was designated as twice the first non-zero frequency component to prevent an infinite value at zero frequency [34]. Afterward, a central spherical volume with a diameter of 380 voxels was extracted from the filtered noise volume to avoid the wrap-around effect caused by the discrete Fourier transform filtering operation [32]. Since the breast anatomy is mostly composed of fibro glandular and adipose tissues [35, 37], we sorted the voxel values of the spherical volume in descending order and set the attenuation coefficient of the fibro glandular tissue to the top ℱ% voxel values (where ℱ represents VGF). The remaining (100−ℱ)% voxel values were assigned the attenuation coefficient of the adipose tissue. In this work, we considered a breast phantom with 30% VGF to investigate signal detectability on the DBT image quality as shown in Fig 1(a) because 30% VGF is more relevant to the composition of real patient breast anatomy [38, 39]. Note that we assigned the attenuation coefficients of the fibro glandular tissue and adipose tissue to be 0.0802 mm^−1^ and 0.0456 mm^−1^, respectively, which are equivalent to the attenuation coefficients at 20 keV monochromatic energy corresponding to the mean energy of the nominal 28 kVp incident spectrum [15, 40].

To model a breast mass, we used a spherical object with a 1 mm diameter as a signal, and replaced the attenuation coefficients of the breast phantom in the signal regions with those of the signals [17, 36]. The attenuation coefficient of each signal was 0.0844 mm^−1^ at 20 keV monochromatic energy [40]. The voxel and volume sizes of the simulated breast phantom were 0.11 × 0.11 × 0.11 mm^3^ and 41.80 × 41.80 × 41.80 mm^3^, respectively.

#### Digital breast tomosynthesis systems

We used the geometry of the DBT system shown in Fig 1(b). The X-ray source was positioned at (0 mm, 0 mm, 605 mm), and the 500 × 500 (*x*-axis × *y*-axis) flat-panel detector array with a 0.125 × 0.125 mm^2^ detector cell size was centered at (0 mm, 0 mm, -45 mm) [17]. The X-ray source and flat-panel detector rotated simultaneously along an arc path within a limited data acquisition angle of ℛ° [15, 41, 42]. Projection data were acquired from *N* views in step-and-shoot mode [19, 43, 44]. To investigate the effect of system geometry on image quality, two different data acquisition angles with different number of projection views were used: 1) ℛ°=20° and *N* = 11, and 2) ℛ°=60° and *N* = 31. Note that projection data were acquired with a 2° sampling interval over the data acquisition angles in both geometries.

The angular projection data of breast phantoms were calculated using a forward projector that computed the radiological path along the ray between the X-ray source and each detector cell [45]. In the discrete-to-discrete projection procedure, the voxel size of the breast phantom is required to be smaller than the cell size of the imaginary detector at the iso-center to avoid discretization artifacts [46]. In this study, the voxel size of the breast phantoms (i.e., 0.11 × 0.11 × 0.11 mm^3^) was smaller than the magnified detector cell size at the iso-center (i.e., 0.1163 × 0.1163 mm^2^), which was sufficient to avoid the discretization artifact. To use the same radiation dose for different data acquisition angles, uniform noise following the Poisson distribution was generated using 2.3 × 10^5^/*N* incident photons per detector cell as quantum noise. Note that using 2.3 × 10^5^ incident photons per detector cell is equivalent to the dose level of 1.6 mGy for a 4 cm breast with 28kVp spectrum, which is the typical dose level for a single-view mammography system [17].

**Fig 2:**
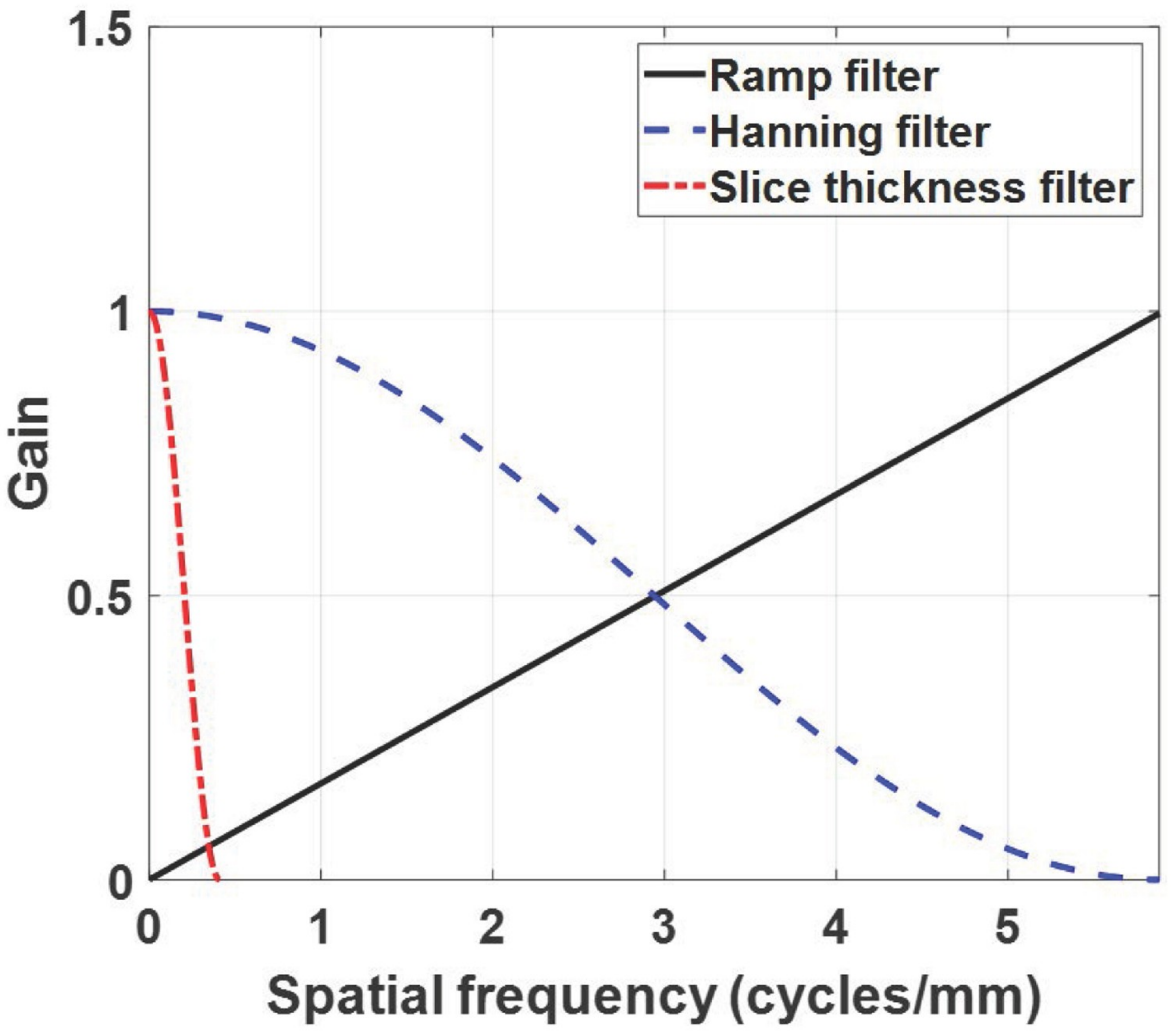
Profiles of the reconstruction filters.

In the FDK reconstruction of DBT system [47], three filters are used, including 1) ramp, 2) Hanning (i.e., one of noise apodization filters) filters applied along the *x*-direction, and 3) ST filter which prevents aliasing artifacts applied along the *z*-direction. The ramp filter is implemented as described in [48], and the Hanning and ST filters used in this study are expressed as [22]

$$\begin{array}{c} F_{\text{hann}}\left(f_{x}\right)=\{\begin{array}{cc} 0.5+0.5\text{cos}(\frac{\pi f_{x}}{f_{NY}}) & \text{for}\;\;|f_{x}|<f_{NY}\\ 0 & \text{elsewhere} \end{array} \end{array}$$ (2)

$$\begin{array}{c} F_{\text{ST}}\left(f_{z}\right)=\{\begin{array}{cc} 0.5+0.5\text{cos}(\frac{\pi f_{z}}{\gamma f_{NY}}) & \text{for}\;\;|f_{z}|<\gamma f_{NY}\;\text{and}\;\left|f_{z}\right|<\text{tan}\left(R^{{}^{\circ}}\right)f_{NY}\\ 0 & \text{elsewhere} \end{array} \end{array}$$ (3)

where *f*_*x*_ and *f*_*z*_ are the coordinate system of the frequency domain along the *x*- and *z*-directions, and *f*_*NY*_ is the Nyquist frequency (i.e., 5.88 cycles/mm). In this work, *γ* was set to 0.07 as a multiplicative factor to avoid aliasing artifacts. Note that the cutoff frequency of the ST filter depends on the acquisition angle ℛ°, and that the degree of image blurring increases as the acquisition angle increases. Fig 2 shows the profiles of the reconstruction filters, and the three different filter schemes used in this work are summarized in Table 1.

**Table 1:** The three filter schemes used in the reconstruction.

Filter scheme	Filter combination
Scheme 1	Ramp filter
Scheme 2	Ramp filter + Hanning filter
Scheme 3	Ramp filter + Hanning filter + ST filter

The noisy projection data were filtered using three different filter schemes, and then voxel-driven back-projection with linear interpolation was performed [48]. The volume size of the reconstructed image was about 32.3 × 32.3 × 64.0 mm^3^ (380 × 380 × 64 array) with a voxel size of 0.085 × 0.085 × 1.0 mm^3^ [17]. Note that reconstructed pixel size of in-plane image (i.e., 0.085 mm) was set to be small enough to avoid noise aliasing. The central in-plane (*x*-*y* plane) images (128 × 128 array) were extracted from the reconstructed breast volume images and used to evaluate the DBT image quality. The simulation parameters of the DBT system are summarized in Table 2.

**Table 2:** Simulation parameters.

Parameter	Value
Source to iso-center distance	605 mm
Detector to iso-center distance	45 mm
Data acquisition angle (${ℛ}^{{}^{\circ}}$)	ℛ°=20° and *N* = 11 (From −10° through 10°)
- Number of projection views (*N*)	ℛ°=60° and *N* = 31 (From −30 through 30°)
Detector cell size	0.125 × 0.125 mm^2^
Detector array size	500 × 500 (*x*-axis × *y*-axis)
Reconstructed volume size	32.30 × 32.30 × 64.0 mm^3^
Reconstructed voxel size	0.085 × 0.085 × 1.0 mm^3^
Reconstructed matrix size	380 × 380 × 64
X-ray energy	20 keV monochromatic energy
Number of incident X-ray photons	2.3 × 10^5^/*N*
Reconstruction algorithm	FDK

### Image quality assessment

#### The human observer study

To evaluate the signal detection performance, we conducted binary detection tasks with signal-known-exactly (SKE) and background-known-statistically (BKS) schemes. The two hypotheses (i.e., *H*_0_ for signal-absent and *H*_1_ for signal-present) are given by

$$\begin{array}{c} H_{0}:g=b_{sa}+n \end{array}$$ (4)

$$\begin{array}{c} H_{1}:g=b_{sp}+n \end{array}$$ (5)

where vector **b**_**sa**_ is the anatomical background, and vector **b**_**sp**_ is the anatomical background containing the spherical signal. Vector **n** is the reconstructed tomosynthesis noise, and vector **g** is the reconstructed in-plane image.

**Fig 3:**
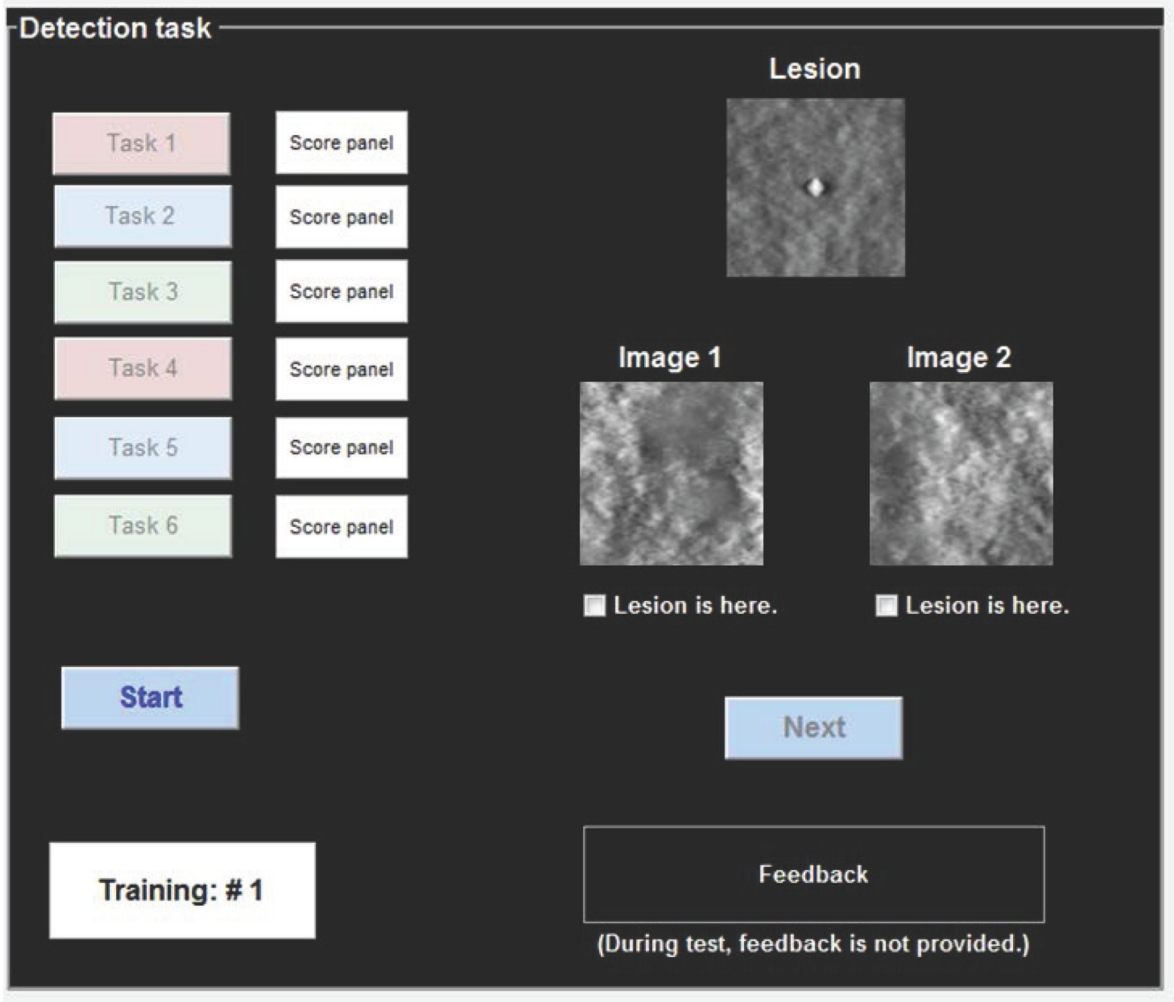
The binary detection task for the human observer study with respect to six detection tasks.

In the human observer study, 10 human observers performed six detection tasks, as reported in Table 3. Each observer simultaneously viewed the signal-present and signal-absent images displayed on a Nio 3MP LED monitor (Barco, Kortrijk, Belgium), and selected the signal-present image shown in Fig 3. We set image display window and level using the mean value and standard deviation of image set, which could not be controlled by the human observer [18]. In each trial, the signal-present and signal-absent images were randomly positioned, and there were no restrictions on decision time and viewing distance.

**Table 3:** Detection tasks for different data acquisition angles and reconstruction filter schemes.

Task	Data acquisition angle	Reconstruction filter scheme
Task 1	ℛ°=20° and *N* = 11	scheme 1
Task 2	ℛ°=20° and *N* = 11	scheme 2
Task 3	ℛ°=20° and *N* = 11	scheme 3
Task 4	ℛ°=60° and *N* = 31	scheme 1
Task 5	ℛ°=60° and *N* = 31	scheme 2
Task 6	ℛ°=60° and *N* = 31	scheme 3

For human observer training, we provided 30 image sets with feedback in each task. Afterward, 100 image sets without feedback were used to test human observer detection performance. Note that training and test image sets were independent for each observer. To evaluate the signal detectability of the human observers, we calculated the percent correct (*P*_*c*_) as follow:

$$\begin{array}{c} P_{c}=\frac{1}{N_{t}}\sum_{s=1}^{N_{t}}o_{s}, \end{array}$$ (6)

where *N*_*t*_ is the number of test trials. If an observer finds the correct image (i.e., signal-present image) in the *s*-th trial, *o*_*s*_ is 1, else it is 0. The variance of *P*_*c*_ was estimated by bootstrapping *o* value 1,000 times [49, 50].

To compare the detection performance of human observers with that of the mathematical model observer, *P*_*c*_ is converted to the task signal-to-noise ratio (SNR_t_) as follows [26]:

$$\begin{array}{c} {\text{SNR}}_{t}=2\times erf^{-1}\left(2\times P_{c}-1\right), \end{array}$$ (7)

where *erf*^−1^ represents the inverse of the error function.

#### The mathematical model observers

We used CHO with two different channels: 1) LG channels (LG CHO) [16–18, 28] to approximate the performance of the Hotelling observer and 2) D-DOG channels (D-DOG CHO) [26, 32] to mimic the properties of the human visual system.

**Fig 4:**
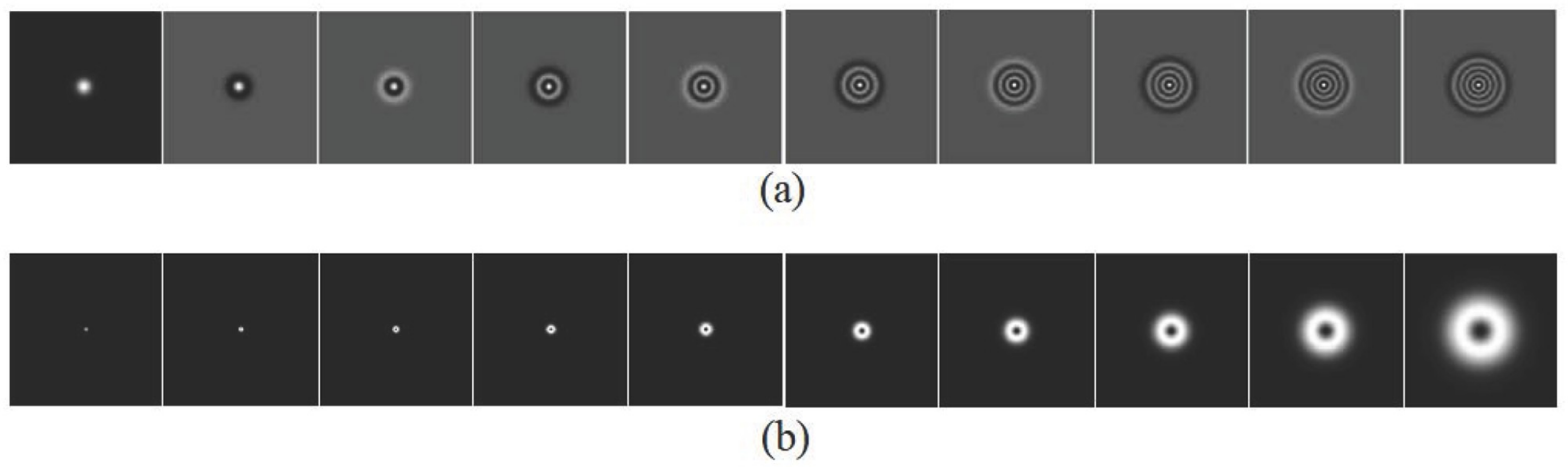
Exampled channels of LG CHO and D-DOG CHO. (a) Exampled 10 number of LG spatial channel images with *a*_*u*_ = 11 pixels (left: *p* = 0, right: *p* = 9). (b) Exampled 10 number of D-DOG frequency channel images (left: *i* = 1, right: *i* = 10).

- *LG channels*: these are generated as Gaussian functions as follows:
  $$\begin{array}{c} u_{p}\left(r|a_{u}\right)=\frac{\sqrt{2}}{a_{u}}\text{exp}(\frac{-\pi r^{2}}{a_{u}^{2}})L_{p}(\frac{2\pi r^{2}}{a_{u}^{2}}), \end{array}$$ (8)
  with the Laguerre polynomial function
  $$\begin{array}{c} L_{p}\left(x\right)=\sum_{k=0}^{p}{\left(-1\right)}^{k}(\frac{p}{k})\frac{x^{k}}{k!}, \end{array}$$ (9)
  where **r** is a 2D spatial coordinate, *a*_*u*_ is the width of the Gaussian function, and *p* is the polynomial order. Note that the Gaussian width *a*_*u*_ should be determined to maximize the corresponding signal detectability of LG CHO to approximate the performance of Hotelling observer. Since the reconstruction process introduces signal blurring by reconstruction filters and data acquisition geometry, even if the signal size is known, we still need to find the *a*_*u*_ that maximizes the signal detection performance for each task by brute-force searching within the range of 3 to 80 pixels. Note that the optimal Gaussian width was proportional to the diameter of the spherical object. We used 20 LG channels to evaluate the detection performance because the detectability of LG CHO saturated when more than 20 LG channels were used. Example 10 LG channel images are shown in Fig 4(a).
- *D* − *DOG channels*: these are defined using multiple bandpass filters, and the *i*-th channel profile is expressed in the frequency domain as
  $$\begin{array}{c} C_{i}\left(\rho \right)=\text{exp}[-\frac{1}{2}{\left(\frac{\rho }{Q\sigma_{i}}\right)}^{2}]-\text{exp}[-\frac{1}{2}{\left(\frac{\rho }{\sigma_{i}}\right)}^{2}], \end{array}$$ (10)
  $$\begin{array}{c} \sigma_{i}=\sigma_{0}\alpha^{i}, \end{array}$$ (11)
  where *ρ* is the radial frequency, *σ*_*i*_ is the standard deviation of each channel, *Q* is a multiplicative factor that defines the channel bandwidth, and *i* is the channel index. The channel parameters given in [32] are *σ*_0_ = 0.005, *α* = 1.4, *Q* = 1.67, and *i* = 1–10. Fig 4(b) shows example images of 10 D-DOG channels. Note that the D-DOG CHO was derived for low resolution images (e.g., nuclear resolution) [32], but many researchers have used the D-DOG CHO as an anthropomorphic model observer to assess breast image quality with high resolution and showed that the detection performance of the D-DOG CHO correlated with human observer’s performance [36, 51–53].

To reduce the data dimensionality of **g**, channelized image **v** can be expressed by applying channel matrix **T** to **g** as given by

$$\begin{array}{c} v=Tg. \end{array}$$ (12)

Matrix **T** of the LG channels is implemented using discrete sample values from Eq (8). For the D-DOG channels, matrix **T** was discrete sample values from the inverse Fourier transform of Eq (10).

CHO template **w**_**v**_ and decision variable *t*_*j*_ can be derived as

$$\begin{array}{c} w_{v}={K_{v}}^{-1}\Delta v_{s}, \end{array}$$ (13)

$$\begin{array}{c} t_{j}={w_{v}}^{t}v_{j},\;\;\;\;\;\;\;\;\;\;\;\;\;\;\;\;\;\;j=0,\;1, \end{array}$$ (14)

where the covariance matrix **K**_**v**_ is computed by averaging the covariance matrices of the signal-present (**v**_1_) and signal-absent (**v**_0_) channelized images, and Δ**v**_**s**_ is the mean difference between the two channelized images. The detectability of model observer is typically higher than that of a human observer, and thus we applied internal noise to D-DOG CHO to match the human observer performance. In this study, we add decision variable internal noise [54] as follows:

$$\begin{array}{c} t_{j}^{\text{int}}=t_{j}+\varepsilon , \end{array}$$ (15)

where variable *ε* indicates internal noise, which is sampled from a normal distribution with zero mean and constant standard deviation (i.e., *N*(0, *p*)).

As a figure of merit for the detection performance, we calculate the SNR_t_ value [25] given by

$$\begin{array}{c} {\text{SNR}}_{t}=\frac{<t_{1}>-<t_{0}>}{\sqrt{\frac{1}{2}\left(\sigma_{t_{1}}^{2}+\sigma_{t_{0}}^{2}\right)}}, \end{array}$$ (16)

where <°> is an expectation operator. Variables *t*_1_ and *t*_0_ are the signal-present and signal-absent decision variables, respectively, and $\sigma_{t_{1}}$ and $\sigma_{t_{0}}$ are the corresponding standard deviations. Note that SNR_t_ of D-DOG CHO with internal noise was calculated using variable $t_{1}^{int}$ and $t_{0}^{int}$ in Eq (16).

For observer training, we estimated the covariance matrix **K**_**v**_ using 500 image pairs, and another 500 independent image pairs were used to calculate Δ**v**_**s**_. For observer testing, decision variables *t*_*j*_ were computed using 100 image pairs, which were independent from training images. The variance of the SNR_t_ was estimated by bootstrapping the decision variables 1,000 times [49, 50]. For model observer variability, we used 10 trained model observers using independently generated training data sets, and then averaged the 10 SNR_t_ values. For the standard deviation of internal noise, variable *p* was selected to minimize the root-mean-square error (RMSE) in the SNR_t_ values between the mathematical model observer and human observer for all tasks.

#### Contrast-to-noise ratio

We computed CNRs [7] using 500 reconstructed in-plane images containing a 1 mm signal. The signal and background regions of interest (ROIs) were defined as 5 × 5 pixel squares centered in the signal and background regions, respectively. The CNRs were computed using the Eq (17) as follows [7]

$$\begin{array}{c} \text{CNR}=\frac{|m_{s}-m_{b}|}{m_{b}\sigma_{b}}, \end{array}$$ (17)

where *m*_*s*_ and *m*_*b*_ are the mean values of the pixels in the signal and background ROIs, respectively, and *σ*_*b*_ represents the standard deviation of the background ROI. Note that the SDNR is basically the same as the CNR, but CNR takes into account the mean value of the background region when calculating the noise part (i.e., denominator part).

#### Signal power spectrum

To show the effect of reconstruction filters on signal power distribution, we computed SPS as a function of data acquisition angle. We reconstructed signal images without anatomical noise for each reconstruction filter and data acquisition angle. To suppress spectral leakage caused by the discrete Fourier transform, the Hanning tapering window given by Eq (18) was applied to each signal image [12, 37].

$$\begin{array}{c} W\left(r\right)=\{\begin{array}{cc} 0.5+0.5cos(\pi r/D) & \text{if}r\leq D\\ 0 & \text{if}r>D \end{array} \end{array}$$ (18)

where *r* is the radial distance from the center, and *D* is half of the image width. The 2D SPS was calculated by taking the square of the absolute value of the discrete Fourier transform of each reconstructed signal image. Then, we performed radial averaging of the 2D SPS to yield the 1D SPS.

#### Exponent *β* value of anatomical noise power spectrum

We compute the ensemble mean value using 500 signal-absent reconstructed in-plane images for each reconstruction filter and data acquisition angle, and subtracte it from each image, yielding zero mean signal-absent images. We apply the Hanning tapering window in Eq (18) to the zero mean signal-absent images to prevent the appearance of artifacts caused by the spectral leakage. The 2D NPS was calculated by ensemble averaging the square of the magnitude of the discrete Fourier transform of each tapered image [55], and radial averaging of the 2D NPS was performed, resulting in 1D NPS. The natural logarithm was applied to the radially averaged 1D NPS to accentuate different noise structures.

To estimate the *β* value in Eq (1), a linear regression was performed on the logarithm-applied 1D NPS over frequency ranges, and the ranges were chosen by maximizing the fit of the linear regression model as determined by the coefficient of determination (i.e., *R*^2^) [12].

## Results

**Fig 5:**
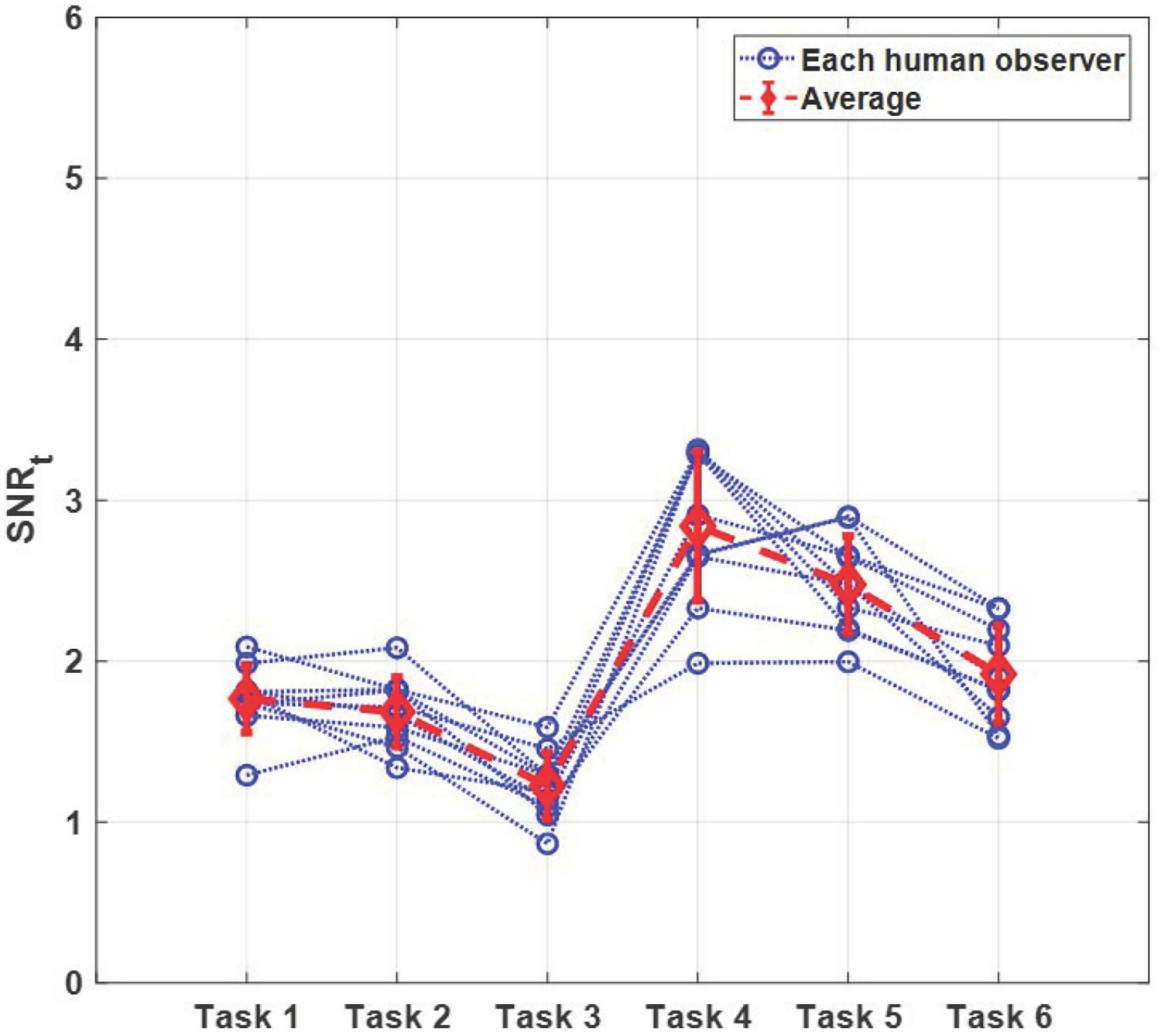
SNR_t_ values of human observer study. The SNR_t_ values of each human observer (indicated by the blue dotted line and circle marker) and averaged SNR_t_ value (indicated by the red dashed line and diamond marker) with 95% confidence intervals for all tasks.

**Fig 6:**
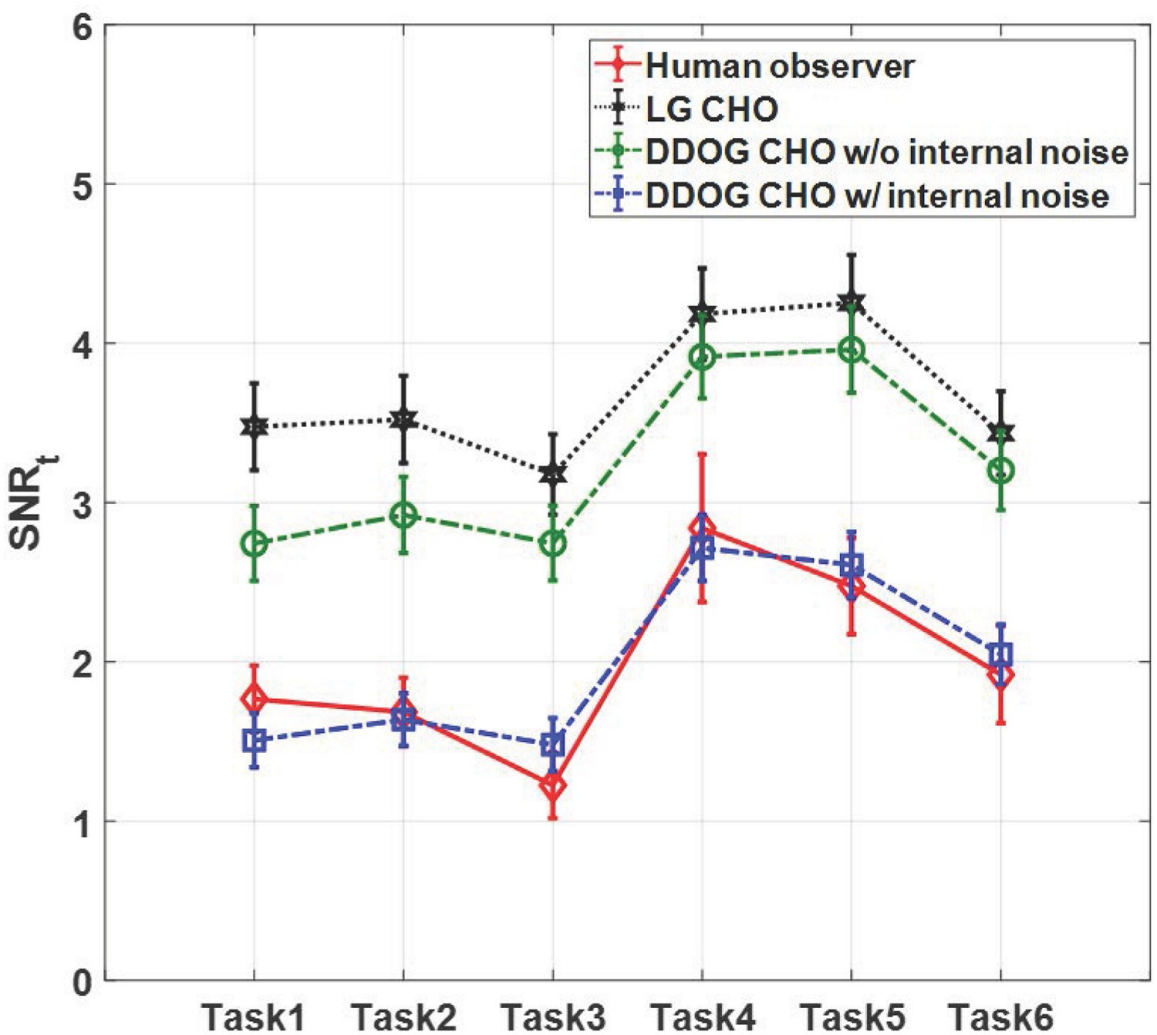
SNR_t_ values of human and model observers. The SNR_t_ values of the human and model observers with 95% confidence intervals for all tasks.

Fig 5 shows the SNR_t_ values of the 10 human observers (indicated by the blue dotted line and circle marker) and averaged SNR_t_ value (indicated by the red dashed line and diamond marker) with 95% confidence intervals for all 6 tasks. Fig 6 shows the SNR_t_ values of the human and model observers with 95% confidence intervals. Additional data acquisition within a larger angular range further reduces overlapping tissue, which improves the signal detectability, as shown in Figs 5 and 6. Note that these trends were also observed in the results of [15, 17], where Hanning weighted ramp filter was used for FBP reconstruction. The SNR_t_ trends of both data acquisition angles (Task 1-3 and Task 4-6) are similar, and the filter scheme 3 yields lower signal detectability than the filter schemes 1 and 2. Although the ST filter is effective to reduce the aliasing artifacts along the *z*-direction, it degrades the signal detection performance of human observer regardless of the data acquisition angles. Note that the the ST filter was used to improve the imaging performance of the DBT system [23] because of its high SDNR, although the SDNR did not reflect the effect of background correlation. Although not presented in this work, we observed similar detectability trends for small signals (i.e., 1 mm and 2 mm) and high fibro glandular density (i.e., 60% VGF). More results are provided in S1 Data.

For all tasks, the SNR_t_ of D-DOG CHO is generally smaller than that of LG CHO because the D-DOG channels suppress the signal energy in the low frequency region [26, 32], but the overall SNR_t_ trends of both model observers are similar, as shown in Fig 6. The SNR_t_ of D-DOG CHO is higher than that of the human observer, which is matched well after including internal noise. Note that the optimal standard deviation for internal noise, *p*, was 4.7 in this work. The Pearson correlation coefficient to quantitatively measure the correlation between detectabilities of human observer and D-DOG CHO with internal noise [56] was 0.95, which indicates that the SNR_t_ values of the human observer and D-DOG CHO with internal noise are well correlated.

Table 4 summarizes the CNR values for all tasks. Since the DBT system used in this work acquires more data sample for the signal as the acquisition angle increases, higher CNRs can be achieved in the reconstructed DBT image with increased data acquisition angle. From the reconstruction filter scheme perspective, filter scheme 2 (scheme 3) introduces background blurring by the Hanning filter (Hanning and ST filters), and thus exhibits a higher CNR than filter scheme 1 due to the reduced background noise. Consequently, the CNR value is the highest when a 60° acquisition angle and filter scheme 3 are used (i.e., Task 6). The CNR trend for the different tasks shows a negative correlation with the human observer performance since it does not reflect the effect of background correlation on signal detection.

**Table 4:** Contrast-to-noise ratio.

Task 1	Task 2	Task 3	Task 4	Task 5	Task 6
29.10	41.90	48.18	38.20	62.86	72.74

**Fig 7:**
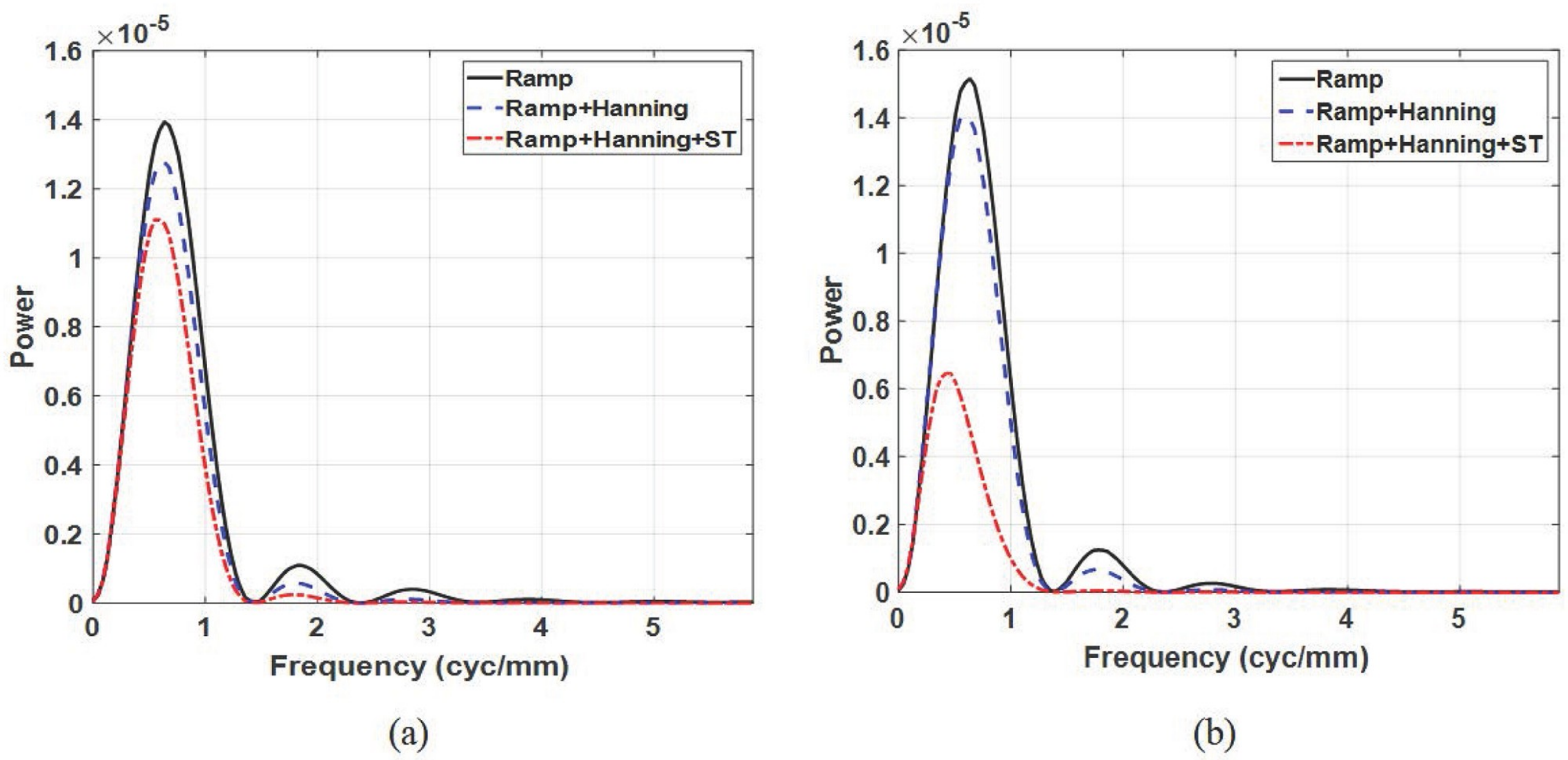
Signal power spectrum. Signal power spectrum for a 1 mm signal diameter with the different reconstruction filter schemes for data acquisition angles of (a) 20° and (b) 60°.

In Fig 7, the SPS is plotted for the reconstruction filter schemes and data acquisition angles. Overall, using a larger acquisition angle can achieve higher SPS because of the additional sampling of the signal for reconstruction. For the reconstruction filter schemes, the filter scheme 2 (scheme 3) degraded the SPS values due to blurring effect of the Hanning filter (Hanning and ST filters) compared to the filter scheme 1. It can also be observed that the SPS of filter scheme 3 for a 60° acquisition angle is much lower than that for 20° acquisition angle because the ST filter makes the signal more blurred as the acquisition angle increases, as described in Eq (3). As a result, in contrast to the CNR results, the filter scheme 1 with 60° acquisition angle achieves the maximum SPS (i.e., Task 4).

**Fig 8:**
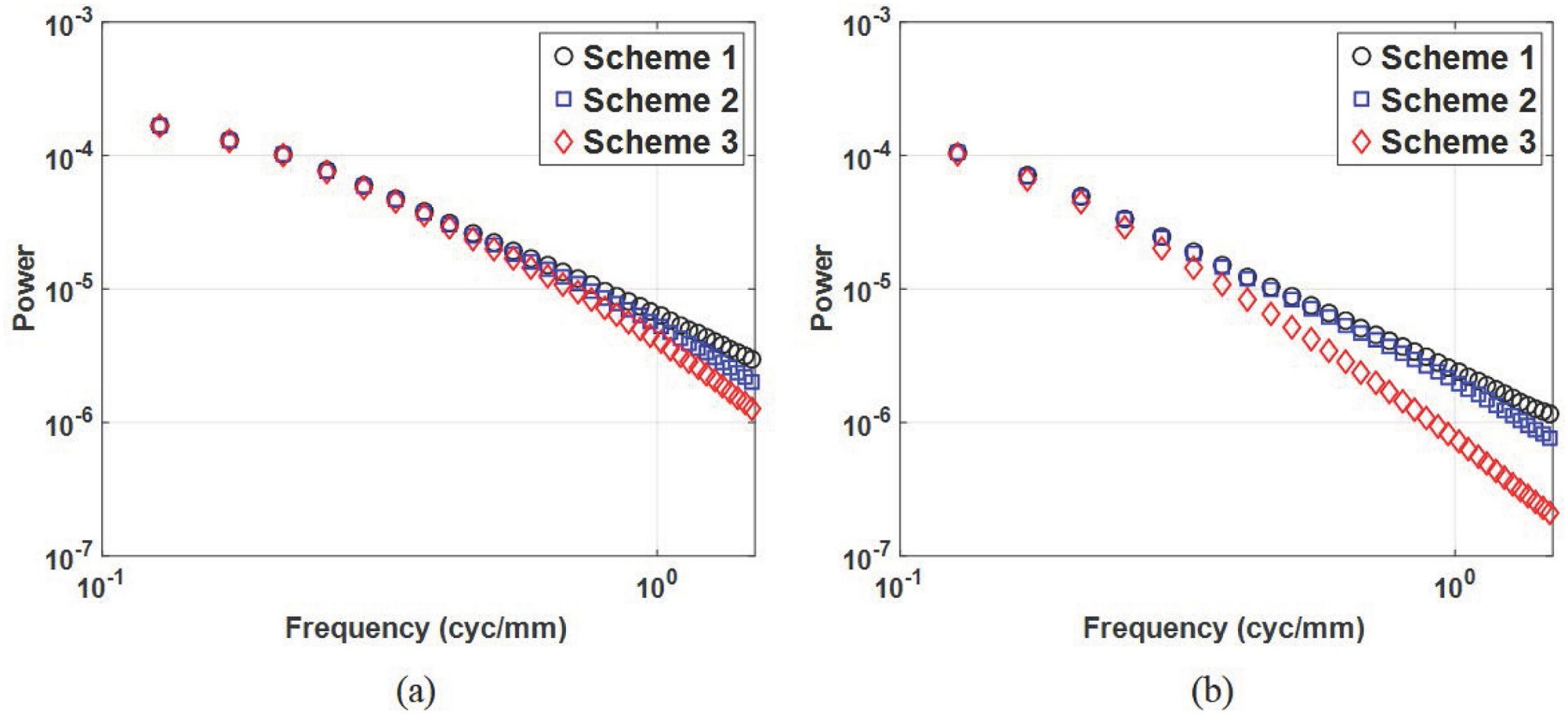
Noise power spectrum. Noise power spectrum from the reconstructed in-plane images for data acquisition angles of (a) 20° and (b) 60°.

Fig 8 illustrates the radially averaged NPS for the reconstruction filter schemes and data acquisition angles. The radially averaged NPS is plotted up to 1.5 cyc/mm because the signal power is concentrated below this value, as shown in Fig 7. As with the SPS, the anatomical NPS from filter scheme 2 (scheme 3) is blurred by the Hanning filter (Hanning and ST filter), and the blurring effect from the ST filter increases when the data acquisition angle increases as shown in Fig 8(a) and 8(b). From the radially averaged NPS, we estimated the *β* values and reported them in Table 5. Note that the fitting frequency ranges was from 0.3 to 0.7 cyc/mm, where the *R*^2^ was larger than 0.99 [37]. Reducing the high frequency energy by reconstruction filter increases the slope of the logarithm-applied radial NPS, which results in a higher *β* values. When the data acquisition angle increases, the overlapping breast tissues are reduced, yielding lower *β* values. However, in the case of filter scheme 3, the noise power blurring by the ST filter is more dominant when the acquisition angle becomes larger, and thus the reduction of overlapping breast tissues does not have a significant effect on the *β* value. From the *β* value perspectives, Task 4 (smallest *β* value) can be regarded as a candidate to optimize the DBT system performance.

**Table 5:** *β* values of the anatomical NPS.

Task 1	Task 2	Task 3	Task 4	Task 5	Task 6
2.02	2.72	3.28	1.83	2.58	3.30

**Fig 9:**
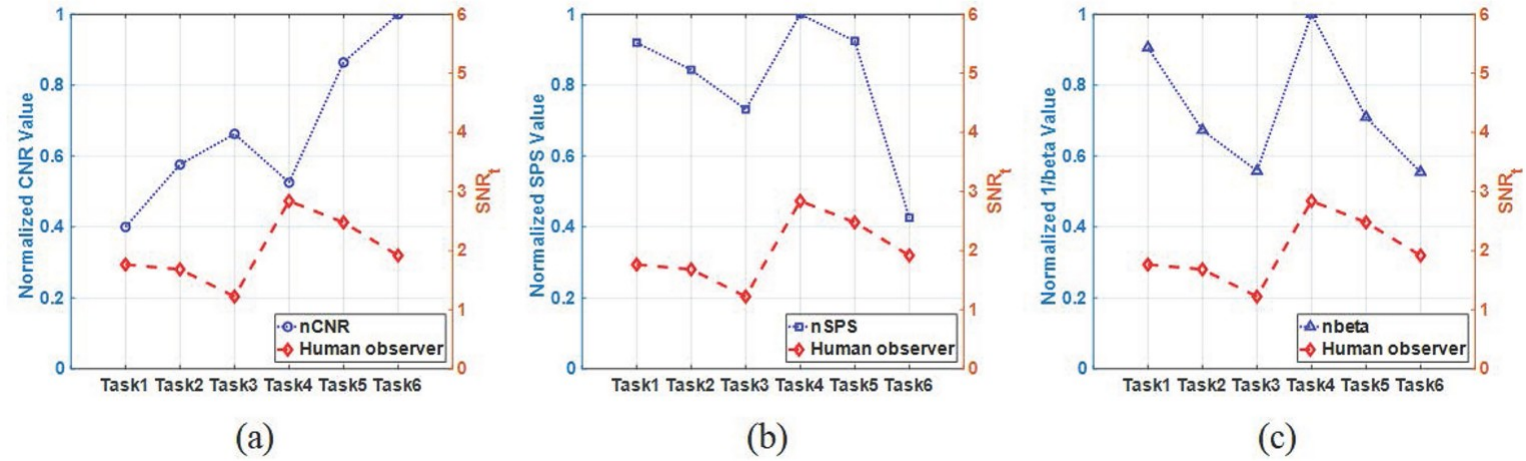
Comparison results between human observer and traditional metrics. Comparison between human observer results and normalized values of (a) CNR (nCNR), (b) SPS (nSPS), and (c) the inverse of *β* value (nbeta).

To investigate the correlation between signal detection performance of human and traditional metrics, we computed normalized CNR (nCNR), SPS (nSPS) and the inverse of *β* value (nbeta), as shown in Fig 9. Note that the nSPS was computed using the peak value of SPS [11], and we calculated nbeta as the inverse of *β* value because a smaller *β* value was regarded as a surrogate with better detectability. Since the background correlation of breast is not reflected in the CNR measurement, the signal detection performance of human observer is not predictable using the CNR metric. In the case of SPS and *β* values, the trends of signal detection performance appear to be similar to detectability of the human observer within the same data acquisition angle (Tasks 1-3 and 4-6) as shown in Fig 9(b) and 9(c). However, it is difficult to expect the relative rank between different data acquisition angles, indicating that either SPS or *β* value is not proper to predict the relative rank of signal detectability for different data acquisition angles in DBT system.

**Fig 10:**
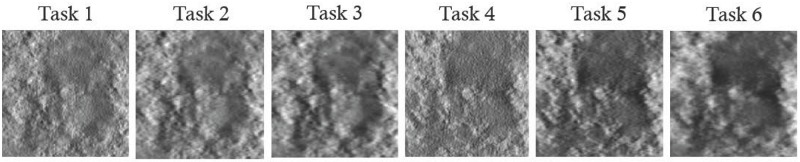
Exampled of reconstructed breast images. Examples of reconstructed in-plane images containing a 1mm diameter signal for all tasks. The display window is set by [min max] mm^−1^ for each task to visualize the background structures more clearly.

For qualitative comparison, Fig 10 shows samples of the reconstructed in-plane breast images used in this work. Reduced anatomical tissue superposition is observed as the acquisition angle increases, which can help improving the signal detectability as predicted from Figs 5 and 6. In Fig 10, it is seen that both the signal and background are blurred by the reconstruction filter, and the degree of the blurring is different across the reconstruction filter schemes. While the aliasing artifact along the *z*-direction is reduced by the ST filter, it is difficult to detect the signal when the ST filter is applied, which is consistent with human and model observers results. Higher CNR, higher SPS, and smaller *β* values were known to correlate with better DBT image quality. However, based on our results in Figs 5–10 and Tables 4–5, these metrics do not represent improved signal detection performance for the in-plane DBT images with various tasks of this study.

## Discussion and conclusions

The main purpose of this work was to investigate the effects of reconstruction filters and data acquisition angles on human observer performance for in-plane DBT images. Our results showed that the detection performance of human observers was dependent on the reconstruction filter schemes in the same data acquisition angle. Despite the advantages of reducing aliasing artifacts, using the ST filter degraded the detection performance of human observers for the signal in the same data acquisition angle. This trend was predicted well using a mathematical model observer with internal noise.

In previous studies [7–14], higher CNR, higher SPS, and smaller *β* values have been considered a surrogate measure for DBT image quality. However, SPS and NPS reflect only half of the numerator or denominator for the Fourier-based ideal observer, and CNR has a limitation to fully reflect of the human perception. For the tasks in this work, the correlation between detection performance of human and the traditional metrics was not observed in both the signal detectability trend and their relative rank.

We used reconstruction filters commonly used in research fields to generate breast tomosynthesis images because it is limited to identify the shapes of reconstruction filters used by commercial vendors due to the confidential properties. To show the effect of reconstruction filters on resolution performance, we measured in-plane modulation transfer function (MTF) along the *f*_*x*_-direction and artifact spread function (ASF) along the *z*-direction. In both data acquisition angles, the in-plane MTF of filter scheme 2 (scheme 3) is degraded by the Hanning filter (Hanning and ST filters) compared to that of filter scheme 1 (S1 Data). And, ASFs are almost the same for the same data acquisition angle regardless of reconstruction filter schemes, which is consistent with the trend observed in a previous study [57], and the spatial resolution along the *z*-direction improved as the data acquisition angle increased (S1 Data). Although we did not use the reconstruction filters used by commercial vendors, we showed that signal detectability was dependent on reconstruction filters. Based on this result, we expect that signal detectability would be changed if the ST filter combined with spectral filters makes the images sharper.

In the FDK reconstruction, projection data were filtered with linear interpolation [*i*.*e*., *sinc*^2^(*f*)] which reduced the high frequency components, and to preserve them, sinc interpolation was often used during back-projection [48]. The frequency responses for linear and sinc interpolations are not significantly different at the low frequency region where the anatomical noise is dominant over the quantum noise for a detection task with signals larger than 1 mm diameter [15, 46, 58], and thus, the trends of signal detectability observed in this study are expected to be similar.

The power spectrum of a large signal is concentrated in low frequency regions where the anatomical noise is dominant over the quantum noise [15, 46, 58]. As shown in S1 Data, the blurring effects by reconstruction filters are more severe in high frequency regions than in low frequency regions. Accordingly, the effect of quantum noise on detectability of a large signal (e.g., 5 mm diameter) is reduced further, and thus, the effect of the reconstruction filters on signal detectability is not noticeable as shown in S1 Data.

To show the effect of sampling intervals on signal detectability, we performed additional experiments with different sampling intervals (i.e., 1° and 3°), and SNR_t_ values were calculated using LG CHO. The overall SNR_t_ trends and levels are similar regardless of sampling intervals (S1 Data), which is consistent with a previous study [17].

In this work, we investigated the image quality of DBT systems through a simulation study. Since DBT systems have many possible configurations that can be optimized, it is essential to conduct investigation using a well-designed DBT simulation including real physical factors. In this study, (1) the blurring effects from the finite focal spot and the detector cell [22] were not considered. These factors introduce additional blurring, but the overall detectability trends would remain the same because the blurring effect from these factors is relatively small compared with that from the reconstruction filters. (2) The effects of X-ray scatter [59] were not considered in the current work because scatter radiation has different effects depending on system geometry, breast thickness, and VGF. To show the effect of scatter radiation on signal detectability, we simulated scatter radiation for DBT systems with the scatter-to-primary ratio (SPR) of 0.475 [60, 61]. The contrast of the reconstructed in-plane images is degraded by scatter radiation, and compared with the detectability without scatter radiation, the overall detectability trends with scatter radiation are similar but the level decreases (S1 Data). (3) Using a sphere object, we evaluated the human observer performance for each task and compared it with its upper performance estimated by LG-CHO. While the LG-CHO can provide optimal detection performance for rotationally symmetric signal shapes, if the signal has more complex shapes with a preferred orientation, LG-CHO shows suboptimal performance. For more complex signal shapes [35, 62], upper detection performance can be estimated using a partial least squares (PLS) channel [63] although it requires much more training dataset. Investigating the detection performance of more clinically relevant signal shapes would be an interesting future research topic.

Since our detection task is relatively simple (i.e., detecting a sphere object with the SKE/BKS schemes), the human observer *P*_*c*_ was close to 1 for tasks 4 and 5 of the 2 mm signal, and all tasks of the 5 mm signal (S1 Data). In actual clinical environments, signal locations, signal shapes, and background statistics are not known. In addition, higher scatter radiation and VGFs degrade the detection performance of larger signals. In this scenario, using a larger signal size would be more appropriate to evaluate the detection performance of human observers.

For the human observer study, 10 human observers were graduate students with individual experiences of over 2 years in human observer experiments. Although human observers were not experienced radiologists, we think that 10 human observers were sufficient number for this work to compare with the model observer because our detection tasks were simple enough, and thus clinical experience would not be necessary. However, for tasks with more complex and clinically relevant diseases, it is desirable to conduct a human observer study with radiologists.

In conclusion, we investigated the signal detectability of the breast tomosynthesis system using different reconstruction filter schemes and data acquisition angles. In contrast to many researchers in the past who thought that reconstruction filters would not affect signal detectability, we found that signal detection performance of the human observers depended on the reconstruction filters in the same data acquisition angle, which is the main finding of this work. To support this finding, we predicted the trends of signal detectability using traditional metrics, which did not predict human perception. In contrast, human observer performance was well predicted by the mathematical model observer, demonstrating its merits in DBT system optimization.

## Supporting information

S1 Data Supplementary data additional investigation results including signal size consideration, 60% VGF breast phantom consideration, in-plane MTF, depth resolution performance, sampling intervals consideration, and scatter radiation consideration. (PDF)
