# Supplementary material for: Human observer performance on in-plane digital breast tomosynthesis images: Effects of reconstruction filters and data acquisition angles on signal detection
Source: PLoS One. 2020 Mar 12;15(3):e0229915. doi: 10.1371/journal.pone.0229915 (PMC7067468; doi:10.1371/journal.pone.0229915)
Supplement: S1 Data — (PDF) [file pone.0229915.s001.pdf]

Supplementary Data

A. Signal size consideration

We investigated the effects of reconstruction filters and data acquisition angles on signal detection using other signal sizes (i.e., 2 mm and 5 mm). As with the 1 mm signal, we replaced the attenuation coefficients of the breast phantoms in the signal regions with those of the signals, and the attenuation coefficient of the signals was 0.0844 mm^{-1} at 20 keV monochromatic energy. Using these phantoms, the in-plane DBT images were reconstructed for all 6 tasks as described in Eq (3) of main manuscript and used to evaluate the DBT image quality.

Fig 1 shows the SNR_t values of the LG CHO with 95% confidence intervals as a function of the signal diameter. Note that the human observer study was not conducted for the 2 mm and 5 mm signals because the P_c values of the human observers were 1 in some tasks (e.g., tasks 4 and 5 for the 2 mm signal, and all tasks for the 5 mm signal). As shown in Fig 1(a), trends of SNR_t values with the LG CHO are similar with the detectability for a 1 mm signal as shown in Fig 6 of main manuscript; in-plane image using filter scheme 3 shows lower detection performance for the 2 mm signal than filter schemes 1 and 2 for both acquisition angles. However, these trends were not observed for the 5 mm signal in Fig 1(b). At the same data acquisition angle, the SNR_t did not show filter scheme dependence due to its larger signal size.

The CNR values are calculated with respect to the signal size and are summarized in Table 1. We observed that higher CNRs can be achieved with larger data acquisition angles. Using filter schemes 2 and 3 introduces background blurring, yielding higher CNR than using filter scheme 1. These observations are similar to the 1 mm signal cases reported in Table 4 of main manuscript. Compared to the CNR of the 1 mm signal, similar CNR values are observed in Table 1 for each task, except for task 6 in

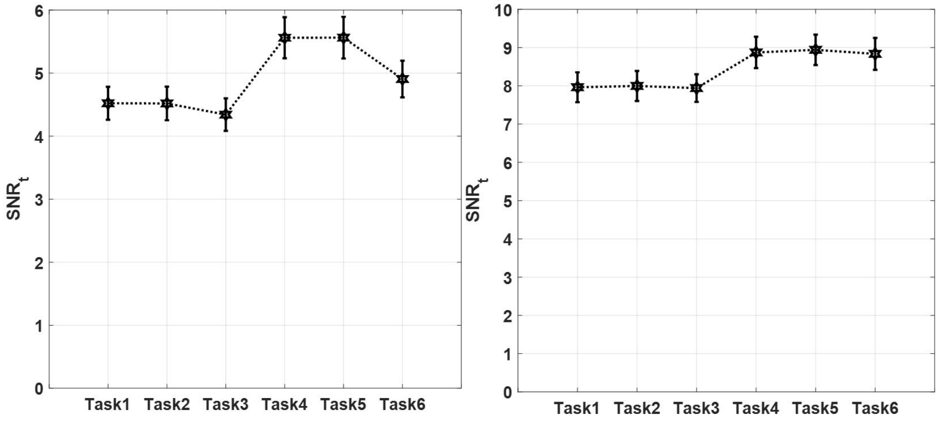

(a) (b)

Figure 1. The SNR_t values of the LG-CHO with 95% confidence intervals for the (a) 2 mm and (b) 5 mm signals.

Table 1. Contrast-to-noise ratio for the 2 mm and 5 mm signals

Signal size	Task 1	Task 2	Task 3	Task 4	Task 5	Task 6
2 mm	24.82	36.96	45.32	37.37	61.51	102.15
5 mm	26.09	38.13	45.69	37.08	60.81	102.24

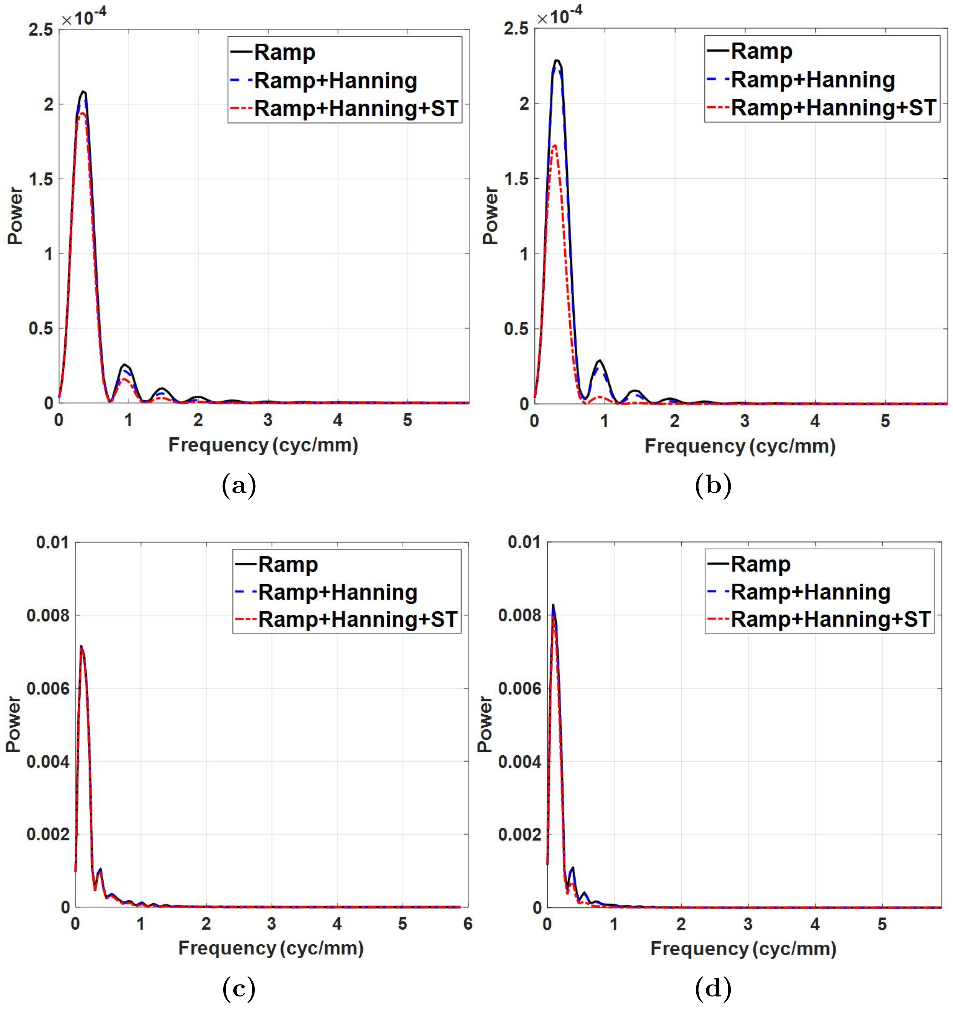

Figure 2. Signal power spectrum for the (a, b) 2 mm and (c, d) 5 mm signals with the different reconstruction filter schemes for data acquisition angles of (a, c) 20° and (b, d) 60°.

which the CNR for the 1 mm signal is lower than that for the 2 mm and 5 mm signals because the effect of signal blurring by the ST filter is more severe in the reconstructed in-plane DBT images than that of other signal sizes. As a result, the mean values of the pixels in the signal ROI (m_s in Eq (17) of main manuscript) become lower, which yields the relatively small CNR value. In Fig 2, the SPS is plotted for 2 mm and 5 mm signals. As shown in Fig 2(a) and (b), filter schemes 2 and 3 degrade the SPS values due to the blurring effect, and the SPS of filter scheme 3 with a 60° acquisition angle

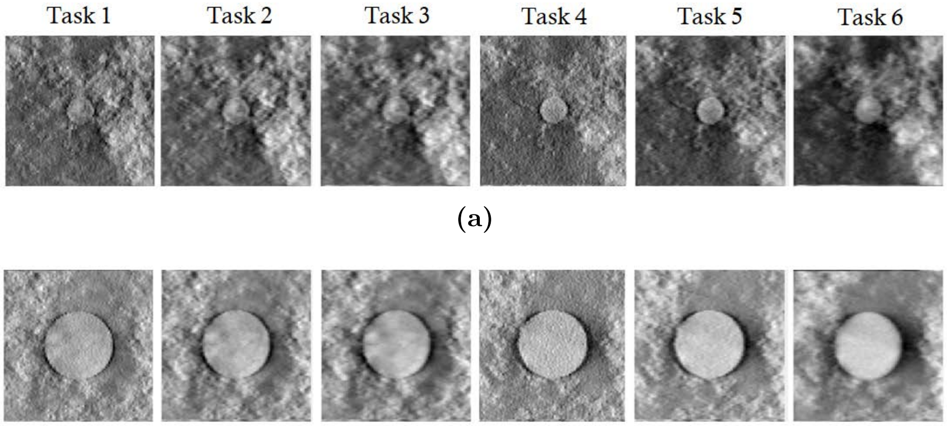

(a)

(b)

Figure 3. Examples of reconstructed in-plane DBT images containing (a) 2mm and (b) 5 mm diameter signals for all tasks. The display window is set by [min max] mm^{-1} for each task to visualize the background structures more clearly.

is much lower than that with 20° acquisition angle because of the ST filter effects, as described in Eq (3) of main manuscript. Although similar behaviors are observed for the 2 mm signal, the 5 mm signal produces almost the same SPS value regardless of the filter scheme because of its larger signal size. For qualitative comparison, examples of the reconstructed in-plane DBT images used in supplementary data A are shown in Fig 3.

In conclusion, although the ST filter is useful to reduce aliasing artifacts along the z-direction, the signal detection performances for the 1 mm and 2 mm signals are degraded, indicating that the choice of reconstruction filter scheme affects the performance in the in-plane DBT images. As mentioned in results section, these results are not predictable using the traditional metrics.

B. Breast phantoms with 60% VGF consideration

To investigate the effect of fibro glandular density on DBT image quality, we considered breast phantoms with 60% VGF (i.e., $\mathcal{F}$=60). The breast phantoms are reconstructed as described in methods section. The $\mathrm{SNR_t}$ values of the LG CHO for the 1 mm, 2 mm, and 5 mm signals are plotted in Fig 4. Since the attenuation coefficient of the signal is similar to that of fibro glandular tissue, the 60% VGFs exhibits a lower signal detection performance compared to the 30% VGF. Similar to the case of the 30% VGF (in Fig 6 of main manuscript), filter scheme 3 yields lower signal detectability overall than filter schemes 1 and 2 for the small signals (i.e., 1 mm and 2 mm). For the large signal (i.e., 5 mm), the signal detection performance is similar regardless of the filter scheme as shown in Fig 4(c).

Table 2 summarizes the CNR values for all tasks and signal sizes. The 60% VGF

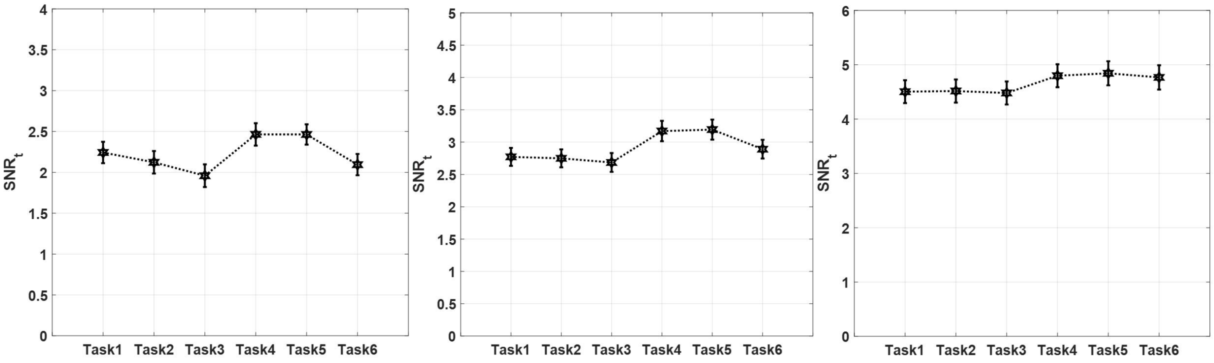

(a) **(b)** **(c)**

Figure 4. The $\mathrm{SNR_t}$ values of the LG-CHO with 95% confidence intervals for (a) 1 mm, (b) 2 mm, and (c) 5 mm signals.

Table 2. Contrast-to-noise ratio for the 1 mm, 2 mm, and 5 mm signals with 60% VGF

Signal size	Task 1	Task 2	Task 3	Task 4	Task 5	Task 6
1 mm	14.94	21.20	24.57	19.66	31.74	35.77
2 mm	12.37	17.83	21.85	19.21	30.93	50.88
5 mm	13.15	19.22	23.53	18.75	30.15	51.95

increases the mean value of the pixels in the background ROI (m_b in Eq (17) of main manuscript), and thus the CNR values are reduced compared to the results with the 30% VGF. Similar to the results in supplementary data A, a higher CNR can be obtained as the data acquisition angle increases because DBT reconstruction recover the signal more accurately with increased data acquisition angle. CNR value is increased more by filter schemes 2 and 3 than filter scheme 1.

Fig 5 shows the radially averaged NPS using reconstructed in-plane DBT images with 60% VGF, and the β values are estimated from the radially averaged NPS as reported in Table 3. Similar β value trends are observed for anatomical backgrounds generated from 30% and 60% VGF. In both cases, smaller β values do not guarantee better signal detection performance in DBT systems; for instance, although the β value of task 6 is higher than that of task 3, the $\mathrm{SNR_t}$ value of task 6 is higher than that of task 3. For qualitative comparison, Fig 6 shows examples of the reconstructed in-plane DBT images used in supplementary data B.

In conclusion, signal detection performance is degraded because of high fibro glandular density, but the trends are similar to the results with 30% VGF. Thus, it is also important to choose an adequate reconstruction filter scheme for signal detection in the presence of high fibro glandular density.

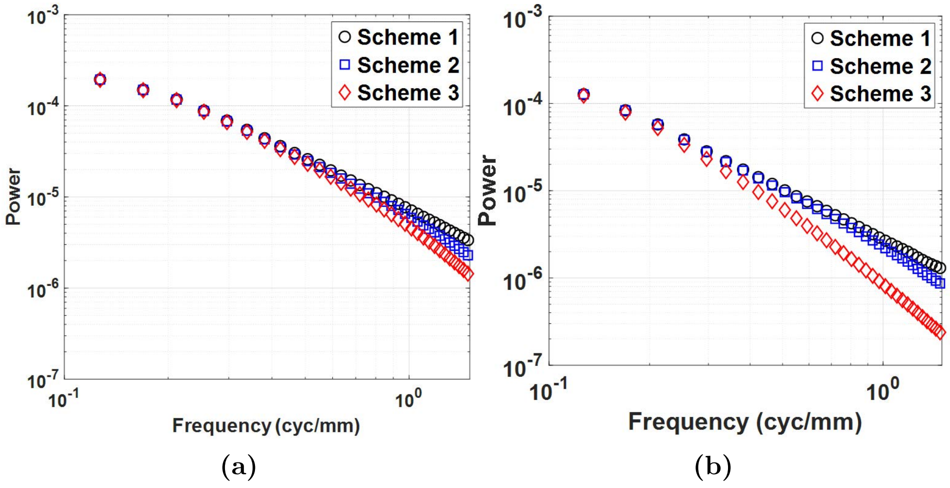

Figure 5. Noise power spectrum from the reconstructed in-plane images with 60% VGF for data acquisition angles of (a) 20° and (b) 60°.

Table 3. β values of the anatomical NPS with 60% VGF

Task 1	Task 2	Task 3	Task 4	Task 5	Task 6
2.12	2.92	3.29	1.88	2.63	3.38

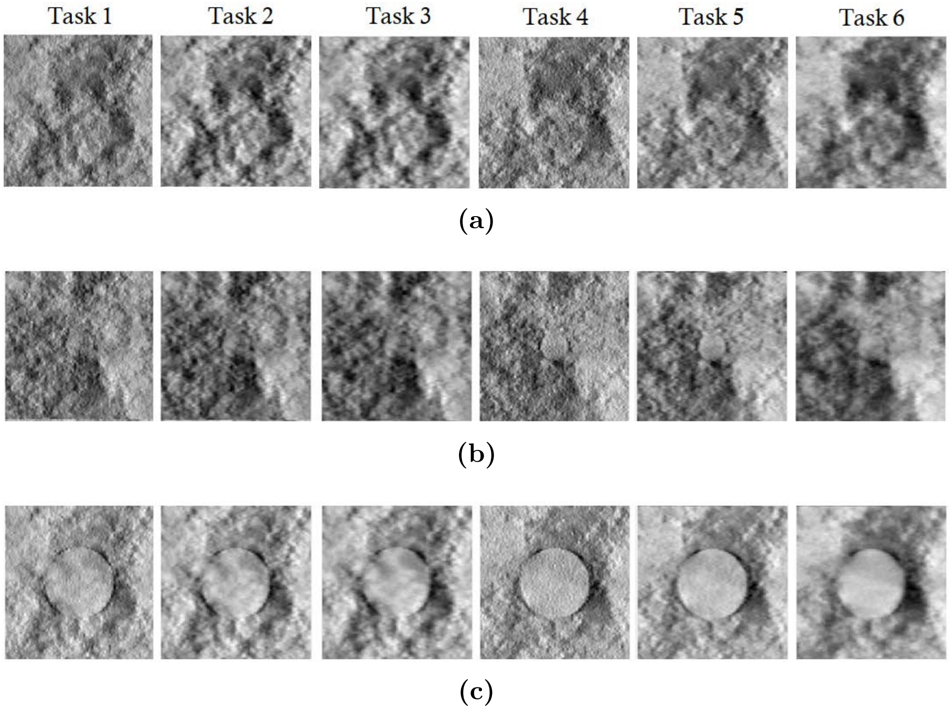

Figure 6. Examples of reconstructed in-plane DBT images with 60% VGF containing (a) 1mm, (b) 2 mm, and (c) 5 mm diameter signals for all tasks. The display window is set by [min max] mm^{-1} for each task to visualize the background structures more clearly.

C. In-plane MTF for all tasks

To show the spatial resolution quantitatively, we measured in-plane modulation transfer function (MTF). Fig 7 shows in-plane MTFs along the f_x-direction for different data acquisition angles, and the in-plane MTFs are plotted up to $2\times f_{NY}$ (i.e., 11.76 cycles/mm). In both data acquisition angles, the in-plane MTF of filter scheme 2 (scheme 3) is degraded by the Hanning filter (Hanning and ST filters) compared to the filter scheme 1. Furthermore, the spatial resolution of filter scheme 3 for 60° acquisition angle is much lower than that for 20° acquisition angle because the ST filter makes the signal and background more blurred as the acquisition angle increases. (Please see the equation (3) in the manuscript.)

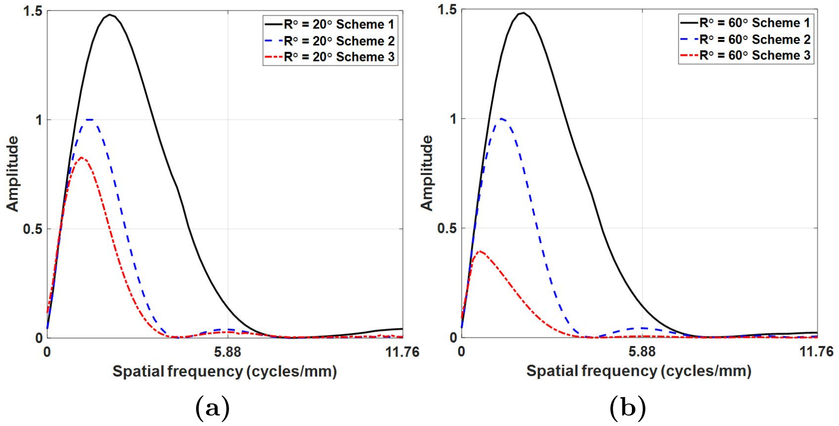

Figure 7. In-plane MTF along the f_x-direction for different data acquisition angles. (a) R° = 20° and (b) R° = 60°. The in-plane MTFs are plotted up to $2\times f_{NY}$ (i.e., 2×5.88 cycles/mm = 11.76 cycles/mm).

D. Depth resolution for all tasks

We calculated artifact spread function (ASF) using the below equation for all tasks to show the depth resolution.

$$ASF(z) = \frac{m_{sig}(z) - m_{bkg}(z)}{m_{sig}(z_0) - m_{bkg}(z_0)}, \tag{1}$$

where z_0 means the location of the in-plane (i.e., in-focus) image along the z-direction. Parameters m_{sig} and m_{bkg} denote reconstructed values of the signal and background, respectively. We calculated m_{sig} and m_{bkg} values by averaging the reconstructed 9 pixels (3×3 pixels) in signal and background regions. Fig 8 shows ASFs for all tasks. It can be seen that the ASFs are almost the same for the same data acquisition angle regardless of reconstruction filter schemes, which is consistent trends observed in [*Zhou 2007 Med.*

Phys.]. In addition, we show that the spatial resolution along the z-direction is improved as the data acquisition angle increases. These results can be seen qualitatively in Fig 9. To show the effect of the different reconstruction tasks clearly, we reconstructed 2 mm diameter signal without anatomical noise. Due to the limited data acquisition angle, shadow artifacts (black region around signal) are visible in the in-plane (x-y) images, and the spreading tails are clearly observed in the x-z images. The spreading tail ranges are dependent on data acquisition angle. Due to the additional data acquisition angle, the spherical signal is more visible in the x-z images when the data acquisition angle is 60° rather than 20°. Since the ST filter makes the signal more blurred as the acquisition angle increases (please see the Fig 7), the signal of filter scheme 3 for 60° acquisition angle (Fig 9 (f)) is more blurred than that for 20° acquisition angle (Fig 9 (c)).

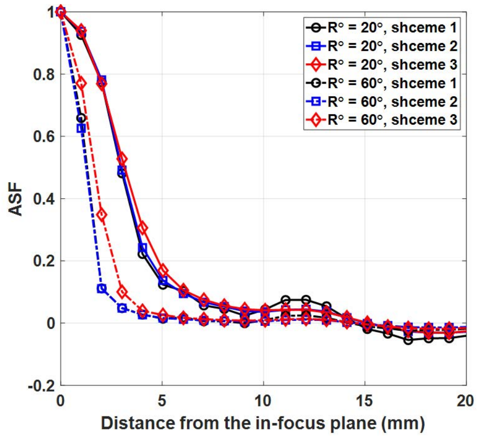

Figure 8. Artifact spread functions for all tasks. The solid line and dashed dot line represent 20° and 60° acquisition angles, respectively. Reconstruction filter scheme 1, 2, and 3 are indicated by circle, square, and diamond markers, respectively.

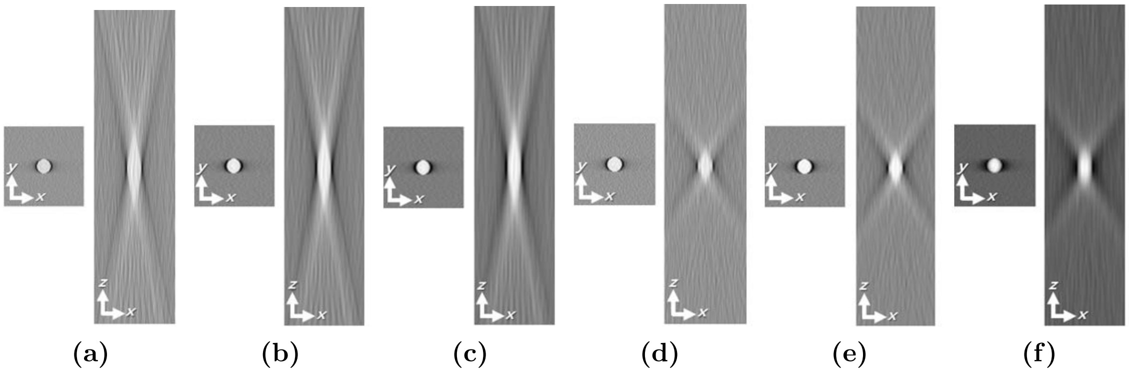

Figure 9. Reconstructed tomosynthesis images, x-y (i.e., in-plane) image and x-z image of the 2 mm diameter sphere object. (a) Task 1, (b) Task 2, (c) Task 3, (d) Task 4, (e) Task 5, and (f) Task 6. The display window is [min max] to show the image characteristics more clearly.

E. Sampling intervals consideration

To show the effect of sampling intervals on signal detectability, we performed additional experiments with different sampling intervals (i.e., 1° and 3°). Additional 12 detection tasks are reported in Table 4. To compare the detection performance of 1° and 3° sampling intervals with that of 2° sampling interval, we calculated $\mathrm{SNR_t}$ using LG CHO following the method described in the manuscript. Fig 10. shows the $\mathrm{SNR_t}$ values for different sampling intervals with 95% confidence intervals. The overall $\mathrm{SNR_t}$ trends and levels are similar regardless of sampling intervals. These results can be expected from previous results [*Zeng 2015 Phys. Med. Biol.*] where they showed that the $\mathrm{SNR_t}$ values were constant over a certain number of views when the same reconstruction algorithm was used.

Table 4. Additional detection tasks for different sampling intervals

Sampling intervals	Task	Data acquisition angle	Reconstruction filter scheme
1°	Task 1	$\mathcal{R}°= 20°$ and $\mathcal{N}= 21$	scheme 1
	Task 2	$\mathcal{R}°= 20°$ and $\mathcal{N}= 21$	scheme 2
	Task 3	$\mathcal{R}°= 20°$ and $\mathcal{N}= 21$	scheme 3
	Task 4	$\mathcal{R}°= 60°$ and $\mathcal{N}= 61$	scheme 1
	Task 5	$\mathcal{R}°= 60°$ and $\mathcal{N}= 61$	scheme 2
	Task 6	$\mathcal{R}°= 60°$ and $\mathcal{N}= 61$	scheme 3
3°	Task 1	$\mathcal{R}°= 20°$ and $\mathcal{N}= 7$	scheme 1
	Task 2	$\mathcal{R}°= 20°$ and $\mathcal{N}= 7$	scheme 2
	Task 3	$\mathcal{R}°= 20°$ and $\mathcal{N}= 7$	scheme 3
	Task 4	$\mathcal{R}°= 60°$ and $\mathcal{N}= 21$	scheme 1
	Task 5	$\mathcal{R}°= 60°$ and $\mathcal{N}= 21$	scheme 2
	Task 6	$\mathcal{R}°= 60°$ and $\mathcal{N}= 21$	scheme 3

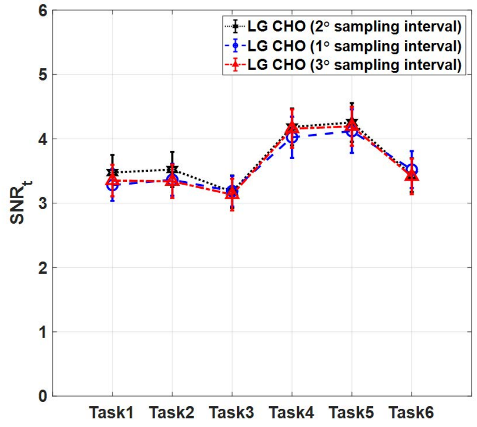

Figure 10. The $\mathrm{SNR_t}$ values of the LG-CHO with 95% confidence intervals for all additional tasks. The similar $\mathrm{SNR_t}$ trends and levels are observed regardless of sampling intervals.

F. Scatter radiation consideration

To show the effect of scatter radiation on signal detectability, we simulated scatter radiation for digital breast tomosynthesis systems with the scatter to primary ratio (SPR) of 0.475 [*Boone 2000 Med. Phys., Sechopoulos 2007 Med. Phys.*]. Fig 11. and Fig 12. show examples of reconstructed in-plane images when the data acquisition angles are 20° and 60°, and the contrast is degraded by the scatter radiation, which causes changes in detectability levels shown in Fig 13. Compared with the detectability without scatter radiation, the overall detectability trends with scatter radiation is similar but the level decreases. In this supplementary data, we used a single SPR value. Since SPR value varies depending on system geometry, breast thickness, and VGF, further investigation with different levels of SPR would be an interesting future research topic.

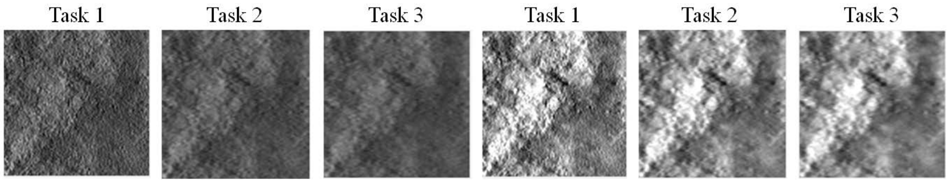

Figure 11. Examples of reconstructed in-plane images (a - c) with and (d - f) without scatter radiation for 20° data acquisition angle. The display window is set by [-0.025 0.12 max] mm^{-1} for each task to visualize the difference due to the scatter radiation.

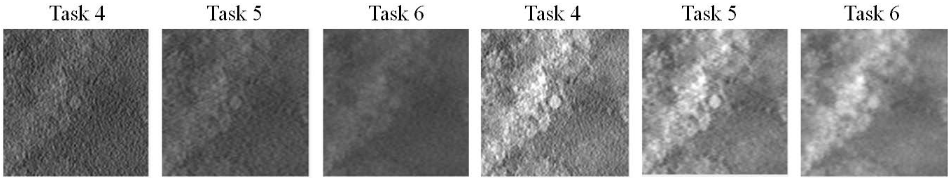

Figure 12. Examples of reconstructed in-plane images (a - c) with and (d - f) without scatter radiation for 60° data acquisition angle. The display window is set by [-0.025 0.12 max] mm^{-1} for each task to visualize the difference due to the scatter radiation.

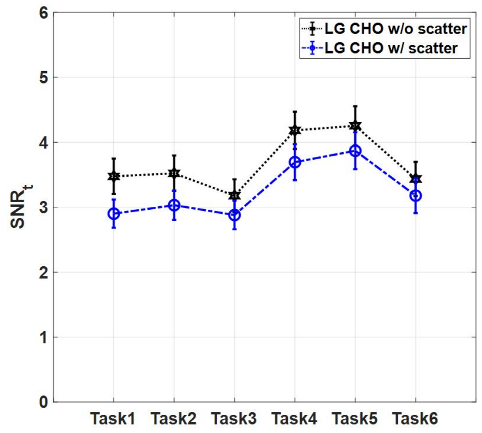

Figure 13. The SNR_t values of the LG-CHO with 95% confidence intervals with and without scatter radiation.
